# A Lower Leg Physical Activity Intervention for Individuals With Chronic Venous Leg Ulcers: Randomized Controlled Trial

**DOI:** 10.2196/15015

**Published:** 2020-05-15

**Authors:** Teresa J Kelechi, Margaret A Prentice, Martina Mueller, Mohan Madisetti, Alexey Vertegel

**Affiliations:** 1 College of Nursing Medical University of South Carolina Charleston, SC United States; 2 Department of Public Health Sciences Medical University of South Carolina Charleston, SC United States; 3 Department of Bioengineering Clemson University Clemson, SC United States

**Keywords:** leg ulcer, physical activity, exercise, mHealth, adherence, randomized controlled trial, feasibility

## Abstract

**Background:**

Individuals with venous leg ulcers (VLUs) suffer disproportionately with multiple chronic conditions, are often physically deconditioned, and demonstrate high levels of physical inactivity.

**Objective:**

The primary objective of this randomized controlled trial was to establish the feasibility of a mobile health (mHealth) physical activity exercise app for individuals with VLUs to improve lower leg function.

**Methods:**

In a 6-week study, adults with VLUs were recruited from 2 wound centers in South Carolina, United States, and enrolled if they were aged 18 years or older with impaired functional mobility and an ankle-brachial index between 0.8 and 1.3. Participants were randomized 1:1 to receive evidence-based, phased, nonexertive physical conditioning activities for lower leg function (FOOTFIT) or FOOTFIT+ with an added patient-provider communication feature. The mHealth Conditioning Activities for Lower Leg Function app also provided automated educational and motivational messages and user reports. Foot movement on the VLU-affected leg was tracked by a Bluetooth-enabled triaxial accelerometer. The study was guided by the Reach, Effectiveness, Adoption, Implementation, and Maintenance framework to assess the feasibility of reach, adherence, acceptability, implementation, and maintenance.

**Results:**

A total of 24 patients were recruited, enrolled, and randomized in the study. Most patients reported difficulty following the protocol for exercising and using the accelerometer and mobile phone and did not use the provider contact feature. However, all patients were adherent to the 6-week exercise program more than 85% of the time for duration, whereas 33% (8/24) of patients adhered more than 85% for the frequency of performing the exercises. Across the three exercise levels, adherence did not differ between the two groups. Confidence limits around the difference in proportions ranged from −0.4 to 0.7. Providers in FOOTFIT+ were inconsistent in checking participant progress reports because of lack of time from competing work commitments. The technology became outdated quickly, making maintenance problematic. Participants said they would continue to exercise their foot and legs and liked being able to follow along with the demonstrations of each level of exercise provided through the app.

**Conclusions:**

The findings of this study suggest that despite initial interest in using the app, several components of the program as originally designed had limited acceptability and feasibility. Future refinements should include the use of more modern technology including smaller wearable accelerometers, mobile phones or tablets with larger screens, an app designed with larger graphics, automated reporting for providers, and more engaging user features.

**Trial Registration:**

ClinicalTrials.gov NTC02632695; https://clinicaltrials.gov/ct2/show/NCT02632695

## Introduction

### Background

Individuals with venous leg ulcers (VLUs) suffer disproportionately with multiple chronic conditions and demonstrate high levels of inactivity [1,2]. Many patients are physically deconditioned and minimally ambulatory, able to only take a few steps at a time, are slow walkers, and have poor standing balance [3]. Obesity, older age, and leg pain are common characteristics that negatively impact functional abilities [4]. Reduced range of motion of the ankle and decreased calf muscle contractility with increased muscle deoxygenation are known physical impairments that also contribute to the worsening condition of the lower legs and substantially further restrict mobility [5]. These processes also contribute to poor wound healing outcomes.

Although numerous study findings suggest physical activity is important for improving outcomes in patients with ulcers, there are inconsistent findings about which types of programs are feasible for functionally impaired individuals with multiple chronic conditions. For those with leg wounds from venous or arterial diseases, enhanced healing and physical and functional abilities are key outcomes of physical activity programs. Evidence from a systematic review of six resistance exercise programs combined with compression therapy, the mainstay of treatment, failed to demonstrate improvements in the proportion of ulcers healed, quality of life, ankle range of motion, and calf muscle pump function [6]. The authors of the review reported the quality of evidence was low because of bias and imprecision of study methodology. Other problems with study methodology include patient and wound complexity, confounders such as comorbid conditions, the need for large samples sizes to show clinically relevant benefits, and the necessary long duration of the trials for wounds to heal, leading to high costs for high-quality trials [7]. However, data from studies of different types of physical activity such as supervised and self-management programs that include aerobic, resistance, and flexibility exercises specific for individuals with VLUs demonstrated high levels of feasibility, safety, and retention of participants [8,9]. Positive outcomes from these studies were noted in physical functions, including walking, sitting to standing, ankle range of movement, wound healing, pain, and lower ulcer recurrence rates. In a study in patients with VLUs, dorsiflexion exercises in which the calf muscle was pumped showed physiological improvements in skin perfusion, including oxygen content and blood flow [10]. Although limited research is available, the mechanisms by which exercises positively influence functional and healing outcomes are posited to be enhanced microvascular circulation through enhanced pumping function of the calf [10].

We previously conducted two small pilot studies of physical activity that informed the development of our study’s intervention methods. The first was a Web-based physical activity intervention developed by our team of physical therapists and exercise specialists that included resistance bands, nonexertive foot movements, and a foot peddler (similar to peddling a bicycle), delivered by a coach (nursing student with degree in exercise science) through online face-to-face internet sessions [11]. A total of 5 participants with venous disease and a history of VLUs participated in determining the feasibility of engaging in three daily doses of nonexertive conditioning physical activities for lower leg function (CALF) for 1 week. We observed a very high level of patient satisfaction with working with the coach and using the equipment to engage in a variety of lower leg exercises while at home. We found the study procedures, including engagement using the Skype (Microsoft Corporation) interface, were feasible. Enhancements were made to CALF, one of which was the addition of a behavioral, motivational interviewing (MI) component, in our second 6-week study of 21 minimally ambulatory patients randomized to the CALF intervention or an exercise handout [12]. Certified wound care nurses were trained on MI communication techniques to interact with patients about engaging in exercises who were receiving wound care in a specialty clinic. We included only the nonexertive foot movements (did not use the peddler or resistance bands in this version of CALF) because of patients having ulcers and their lower legs being wrapped with multilayer compression that restricted ankle movement. The CALF intervention was found to be feasible and acceptable by both patients and the nurses who delivered it. However, having a coach and using providers who require special training are costly and not always available outside the clinic setting, as not all patients receive care in specialized wound clinics. Furthermore, many patients do not have access to physical therapy specialists or readily available transportation to attend exercise programs in the community, and because of having wounds and or functional impairments, many patients find it difficult to engage in physical activity.

### Objective

To address these barriers, technology-enhanced mobile health (mHealth) physical activity interventions are potential solutions to mitigate these barriers, as they have evolved for individuals with chronic conditions such as chronic obstructive pulmonary disease and provide availability for those with limited access to exercise programs [13,14]. In particular, accelerometer-based physical activity programs that provide feedback on steps taken, calories burned, and other physiologic data such as heart rate have become commonplace. Studies suggest that patients with chronic conditions want to participate in physical activity and would consider using an mHealth app if initiated through a clinic or medical office visit [15]. However, few studies integrate accelerometers with apps that routinely engage chronically ill patients and providers in a communication loop about progress toward goals and that are designed to promote adherence to physical activity [16,17].

To address this need, our team developed and tested a foot-based Bluetooth-enabled acceleration tracking (BEAT) device and mobile phone application system and demonstrated its reliability and validity in laboratory experiments using a standard rotary-shaker test with 4 accelerometers (coefficient of variation was found to be 0.7%) and tested its feasibility in minimally ambulatory patients with venous disease and deconditioned legs [18]. The BEAT device detected even very minimal toe movements, which were captured by an app developed for mobile phones that received information from the accelerometer. The primary aim of this small randomized controlled trial study was to explore the feasibility of an exercise program comprised of an accelerometer-app combination, initiated during wound clinic visits, and performed by patients with VLUs in their homes.

## Methods

### Overview

This 6-week randomized clinical trial was designed to test the feasibility of a real-time, nonexertive, lower leg physical activity mHealth program, FOOTFIT, that combined BEAT and CALF. FOOTFIT was compared with FOOTFIT+ comprising BEAT, CALF, and connectivity to a wound clinician via text messaging, phone, or email. Although the primary goal was to establish feasibility, the intervention also targeted the function of the lower legs of individuals with VLUs. The trial complied with the Consolidated Standards of Research Trials (CONSORT) of Electronic and Mobile Health Applications and Online Telehealth guidelines [19], was approved by the Medical University of South Carolina institutional review board (#00043451), and was registered with ClinicalTrials.gov (NCT02632695) on December 17, 2015. Written informed consent was required to participate and obtained before enrollment in the study in which two visits occurred, one at baseline and one at 6 weeks. Participants received US $75 as compensation for participating in the study.

### Recruitment

Participants were recruited through direct referral from two participating wound clinics in the Southeastern region of the United States. Inclusion criteria were being aged 18 years and older, having a VLU, ankle-brachial index of 0.8 to 1.3 to rule out arterial insufficiency, receiving at least weekly wound care anticipated to last for at least 6 weeks from start of the study, being able to don the slipper onto which BEAT was affixed or having assistance from other, and being capable of using a mobile phone (individual observed using his or her phone after enrollment at baseline). Individuals were excluded if they had a comorbid condition such as stroke or severe arthritis that limited ankle function; an ulcer from other causes such as arterial, surgical, or traumatic; cognitive impairment determined by less than 3 recalled words and abnormal clock drawing on the MiniCog test [20] administered at baseline; or no 3G service available where the participant resided. Recruitment goals were established based on state population statistics according to US Census Bureau 2010 data: 3.86% (174,680/4,525,264) ethnic minorities, 28.52% (1,290,684/4,525,364) black, and 67.62% (3,060,000/4,525,364) % white.

### Sample Size and Randomization

The main outcome of this study was feasibility, and the sample size was determined based on pragmatic reasons. The aim was to recruit 24 participants who were randomized to FOOTFIT or FOOTFIT+ in a 1:1 ratio after baseline data collection. A computer-generated random number schema was developed by the statistician who had no contact with the participants. Group allocation was concealed in a database and revealed to the data collector after baseline data were collected and entered to minimize selection and measurement bias. Participants were then informed of allocation to either FOOTFIT or FOOTFIT+.

### FOOTFIT and FOOTFIT+ Interventions

The CALF program for this study consisted of 3 levels of phased, nonexertive movements for the lower legs, beginning with the most minimal level 1 intensity with foot on the floor progressing to level 2 intensity with heal on the floor and forefoot elevated, to maximal level 3 intensity with foot off the floor. Participants were instructed to perform each level for 2 weeks, 3 times per day for a minimum of 15 seconds each per activity, advancing in frequency and intensity. The BEAT was worn on the foot, affixed to a special slipper, during CALF to capture frequency and intensity of movements. The app on the mobile phone reminded individuals to perform the exercises at preset times each day, per patient preference, and sent patients supportive feedback after each daily session. There were also 12 short video clips of how to perform each movement and 16 short audio-recorded, evidenced-based information sessions about managing venous disease, the latest development in ulcer treatment, and other topics of interest expressed by individuals in our previous studies such as why compression is needed, what medications help healing, and how best to elevate the legs to reduce edema.

FOOTFIT+ was enhanced with an added phone, email, or text messaging connectivity feature to the wound care providers. The providers were instructed by the study principal investigator on theory-based patient-provider *talk* communication [21]. The providers were to make weekly contact with the participant via phone, email, or text, per participant preference, and provide a brief report of progress toward goals. If the prescribed activity was not being performed for 2 consecutive days by the participant, the providers were notified via text and reminded to contact the patient to verify the accelerometer was functioning and discuss any problems. The providers were informed that participants could contact them during normal business hours using any mode (phone, email, text, or voicemail), but the communication was to be related to the physical activity program only, such as pain during CALF and progress toward meeting goals. The quantity and quality of the interactions and whether the modes of communication were sustainable in actual practice were assessed. Participants in this group were also made aware that the providers would receive weekly reports about their progress.

### Measures and Outcomes

Data were collected preintervention at the baseline visit and at postintervention during the last visit week. Demographic information included health history, comorbid conditions, ulcer history, medications, age, sex, race/ethnicity, and rural/urban residence. Intervention feasibility assessment was guided by the RE-AIM framework for reach, effectiveness, adoption, adherence, acceptability, implementation, and maintenance [22,23]. Reach was measured by continuous progress monitoring of sample representativeness, how patients learned about the study, types of recruitment activities and rates, meeting of recruitment goals (1 of 5 patients approached, and eligible would be consented), and number of eligible patients approached, consented, and oriented to the study. These data were captured on tracking forms, and quality checks were performed weekly and discussed at weekly team meetings. Data captured by the accelerometer BEAT was reported as the percentage of participants adherent to exercise duration defined as performing foot exercises at or above the recommended duration of 15 seconds or greater for each exercise (3 exercises per session, 3 times per day) or not always adherent (moved foot <15 seconds per exercise per session). Adherence to frequency was reported as the percentage of days the participants completed each CALF intensity level (3 levels performed 3 times per day, ie, 9 levels, each performed over 2 weeks); 9 levels performed 85% of the days or greater throughout the study period was considered adherent to frequency. Participants were instructed to perform the exercises 3 times daily, increase the duration of time each subsequent day, and review additional information on the app as needed. The number and reasons for dropouts were also recorded. Adoption focused on patient-provider communication and was measured by review of 10% of calls/emails/texts between participant and provider via content analysis of communication interactions (ie, information sought, reassurance given, and call was of a social nature). We also assessed whether the provider was checking progress reports and graphs and how the participant rated communication; this information was recorded on tracking forms and assessed at weekly team meetings. The goal for the provider was to use the *talk* model 90% of the time, and that 90% of participants would report high satisfaction. Acceptability was defined as an endorsement and measured by the number and types of problems encountered, such as difficulty using accelerometer and app, and satisfaction with the communication system. Implementation procedures included participant and wound care provider recommendations used to refine CALF or BEAT and the number and types of refinements made. Maintenance was defined as the number of patients who would continue the intervention, and the provider perception of impact and potential for future apps. Safety was evaluated by recording any adverse effects or safety issues such as cramping or new leg pain that occurred during the study period. Treatment fidelity was monitored weekly and assessed retrospectively from data obtained from BEAT in terms of the number of daily exercises completed per participant over the length of the study.

### Statistical Analysis

The study sample was characterized using descriptive statistics. Primary continuous feasibility outcome measures were adherence and acceptability and patient-physician interactions. Additional quantitative measures of feasibility included recruitment and dropout proportions and patient-provider communication measures (ie, clarity of reports, endorsement, potential for adoption, usability of BEAT, and mobile phone). For the continuous measures such as total number completed daily CALF exercises per level, means and medians were obtained. Owing to the small sample size, normally distributed data were not assumed. No hypothesis testing was carried out, and therefore, no *P* values are provided in concordance with recommendations in the CONSORT 2010 statement for randomized, pilot and feasibility trials [24]. Similarly, no effect sizes (eg, Cohen *d*) were provided because of large imprecision with small sample sizes [25]. Instead, 95% CIs for differences in medians between groups were obtained using quantile regression, whereas differences in proportions of categorical feasibility outcomes with their corresponding 95% CIs were obtained using Newcombe risk difference method, to describe estimates of the magnitude of the clinical effects. The technology (problems and acceptability) was evaluated through the documentation on tracking forms and iterative processes using participant interviews. All data were entered on a password-protected Web-based data management system Research Electronic Data Capture and analyzed using SAS software version 9.4 (2016; SAS Institute Inc, Cary, North Carolina).

## Results

### Demographic Data

Age, sex, comorbid conditions, medications, and residence (rural/urban) and other population characteristics are shown in Table 1.

The sample was predominantly white, female, and obese with an average ulcer age of greater than 4 months duration and ulcers occurred most frequently on the lateral and medial aspects of the lower extremities. Several differences were noted in age, employment, education, and marital status, but because of the small sample size, these differences were not assessed for significance or their relationship with feasibility outcomes.

**Table 1:** Demographic and clinical characteristics by FOOTFIT and FOOTFIT+ groups at baseline (N=24).

Characteristic		FOOTFIT (n=12)	FOOTFIT+ (n=12)
Age (years), mean (SD)		60.7 (13.7)	69.1 (11.5)
**Sex, n (%)**			
	Females	8 (67)	6 (50)
**Race, n (%)**			
	Black/African American	4 (33)	6 (50)
	White	8 (67)	6 (50)
**Ethnicity, n (%)**			
	Non-Hispanic/Latino	12 (100)	12 (100)
**Marital status, n (%)**			
	Never married	2 (17)	3 (25)
	Married	7 (58)	3 (25)
	Separated	0	2 (17)
	Divorced	2 (17)	1 (8)
	Widowed	1 (8)	3 (25)
**Educational level, n (%)**			
	Eighth grade or less	0	1 (8)
	Some high school	2 (17)	1 (8)
	High school graduate	2 (17)	3 (26)
	Some college	5 (41)	2 (17)
	College graduate	2 (17)	5 (41)
	Post graduate and/or higher level degree	1 (8)	0
**Employment, n (%)**			
	Employed fulltime	1 (8)	2 (17)
	Not employed	5 (42)	1 (8)
	Retired	6 (50)	9 (75)
**Job classification, n (%)**			
	Professional	4 (33)	5 (42)
	Technical	1 (8)	2 (16)
	Manual	7 (59)	5 (42)
**Residence, n (%)**			
	Urban	5 (42)	9 (75)
	Rural	7 (58)	3 (25)
Weight (lb), mean (SD)		292.5 (104.0)	235.0 (58.9)
BMI (kg/m^2^), mean (SD)		45.2 (14.5)	35.5 (8.8)
**VLU^a^** **location, n (%)**			
	Proximal	1 (8)	1 (8)
	Distal	4 (33)	4 (33)
	Medial	6 (50)	3 (25)
	Lateral	9 (75)	8 (67)
	Anterior	4 (33)	2 (17)
	Posterior	0	2 (17)
Recurrent VLU (yes), n (%)		10 (83)	5 (58)
Age of ulcer (days), mean (SD)		1050.7 (1101.4)	813.8 (928.8)
Age of ulcer (months), mean (SD)		35.0 (36.7)	27.1 (31.0)
Number of ulcers, mean (SD)		7.2 (4.9)	5.2 (9.8)
**Comorbid conditions (top 5), n (%)**			
	Hypertension	9 (75)	7 (58)
	Arthritis	8 (66)	6 (50)
	Diabetes	4 (33)	6 (50)
	Thyroid problems	6 (50)	2 (17)
	Varicose veins	2 (17)	4 (33)
	Vein stripping	4 (33)	2 (17)
**Medications (top 5), n (%)**			
	Antihypertensive med	9 (75)	6 (50)
	Cholesterol	6 (50)	6 (50)
	Pain pills	7 (58)	3 (25)
	Diabetes pills	4 (33)	5 (42)
	Diuretics	4 (33)	4 (33)

^a^VLU: venous leg ulcer.

### Feasibility Outcomes

#### Reach

**Figure 1:**
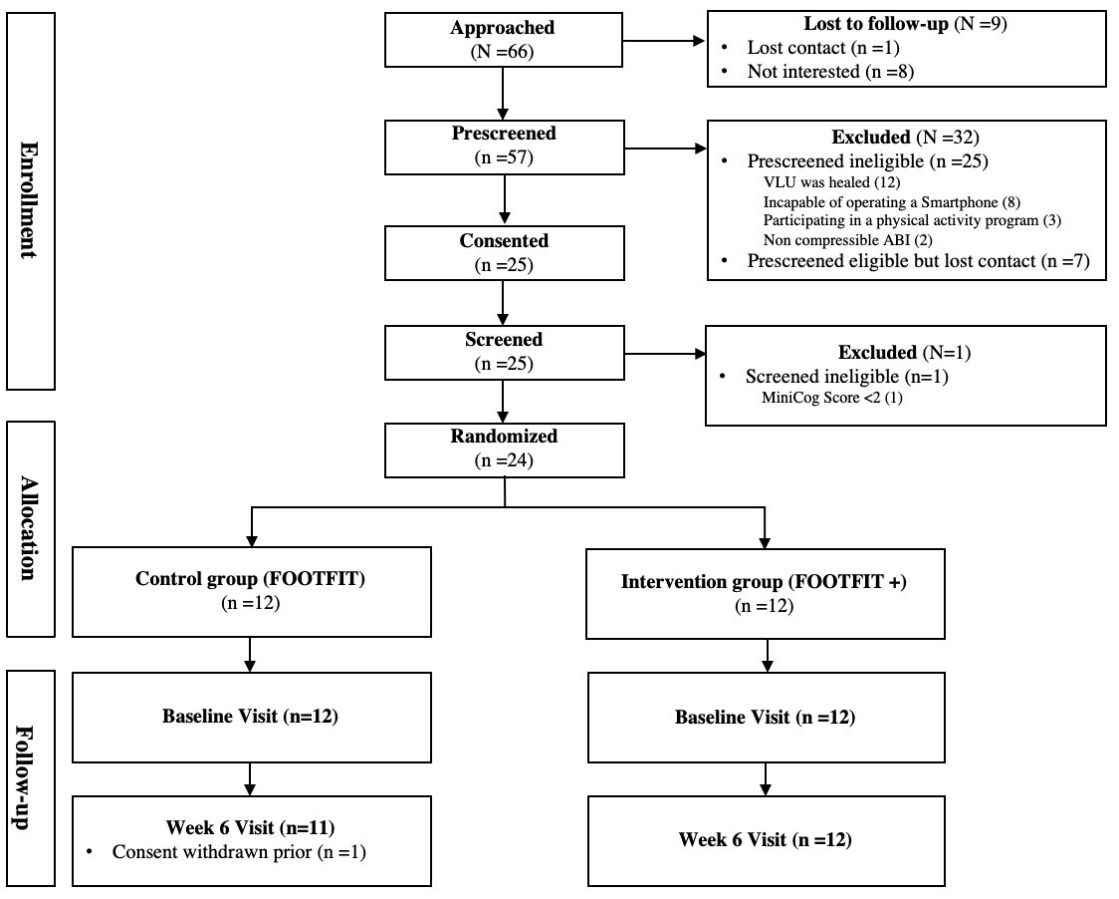
Participant Consolidated Standards of Research Trials flowchart.

Reach data are shown in Figure 1 as the number of patients approached, prescreened eligible, consented, screened, and enrolled.

The sample was representative of the target population except for lacking one Hispanic/non-white participant. Although flyers were posted around clinics and presentations were made by study staff at various venues such as senior living residences and health fairs, all participating in the study were recruited by referral of wound providers in clinic settings. One recruitment goal was to consent and enroll 1 out of 5 patients approached; however, we were unable to track this because of providers’ lack of consistent documentation on study logs. There were no reported adverse events associated with the intervention.

#### Adoption

Patient-provider communication was the focus of adoption; however, none of the participants contacted their provider during the study. Similarly, none of the 3 providers reviewed progress reports and graphs citing time as the major limitation.

#### Acceptability

Acceptability was considered low. The participants encountered several problems with BEAT, the phone, and the app including difficulty remembering to turn BEAT and the phone on and off before and after performing CALF; most participants forgot to close the app at the end of the day, which was needed to reset it to capture data from the next session. The button on BEAT was small and almost flush with the device, making it hard for participants to see and feel to turn it on and off. The phone screen (iPhone 4) was small and challenging for individuals to view. Many participants reported that having to double click the home screen button to close the app was tricky because of arthritic fingers or long fingernails. The app did not work well when individuals wanted to replay the introduction video, and despite attempts by the programmer, this problem could not be resolved. Owing to participants’ ulcer treatments with multilayer compression wraps applied from the toes to the knee, the slippers on which BEAT was affixed did not fit well, and the device sometimes popped off during use. Participants reported that they were *annoyed* by the overly frequent (at a minimum 3 times per day) reminder text or email alerts to start the exercises. If participants were unable to do the foot exercises at that time, the reminder would continue to sound. We observed that although participants demonstrated proficiency during return demonstration of using the accelerometer and app during the baseline visit, several participants became confused when they returned home, prompting calls to study staff for questions although instructions were available in the app.

#### Implementation

Refinements to implementation were mainly related to the app. The app was designed for iOS 8 or later, developed on Heroku cloud platform, and beta tested through Apple TestFlight. Every 3 months, the app’s beta testing period expired, and a new build of the app had to be uploaded by the developers to TestFlight for actively using the app. At these times, the app also had to be updated and reinstalled, often leading to participant confusion and the inability to perform the leg exercises. The providers had no specific recommendations for refinements to reports of participant involvement other than they were too busy to review progress on a regular basis. In response to this challenge, study staff sent the reports each week to the provider, rather than the provider having to access the app site database to search and review data for each participant. The providers did not communicate feedback *back* from the reports to participants. Providers reported they inconsistently reviewed these reports if at all.

#### Maintenance

Participants indicated they liked FOOTFIT, but because of many technical glitches, they believed they would rather do the exercises on their own and not rely on the system. Providers reported that participants told them the exercises helped relieve stiffness and lower leg pain and *enjoyed being in the study*. However, they also revealed to the providers that it was often difficult to operate the accelerometer and phone, corroborating the information reported to study staff.

#### Adherence

Duration and frequency of BEAT use to perform CALF is shown in Table 2. The duration was mostly similar between the two groups; however, adherence to the recommended frequency of use 3 times per day was lower in the FOOTFIT group (17%) than in the FOOTFIT+ group (50%).

**Table 2:** Distribution of adherence level to recommended exercise duration by FOOTFIT and FOOTFIT+ groups (N=24).

Adherence			FOOTFIT (n=12)		FOOTFIT+ (n=12)	
**Duration of exercising (≥15 seconds), n (%)**						
	85%-89%			1 (8)		1 (8)
	90%-94%			2 (17)		0 (0)
	95%-99%			5 (42)		6 (50)
	100%			4 (33)		5 (42)
**Frequency, n (%)**						
	**Always adherent (** **≥** **85%)**					
		85%-89%	1 (8)		1 (8)	
		90%-94%	0 (0)		3 (25)	
		95%-99%	1 (8)		1 (8)	
		100%	0		1 (8)	

#### Exercise Frequency

During weeks 1 and 2 (CALF exercise level 1 intensity), across the two groups, 33% (8/24) of the participants were adherent (moved foot at or above recommended duration of ≥15 seconds per exercise) during all exercises at each session (Table 3).

During weeks 3 and 4 (CALF exercise level 2 intensity), 59% (13/22) of the total sample were always adherent with fewer (3/11, 27%) not always adhering in the FOOTFIT group compared with 55% (6/11) in the FOOTFIT+ group. Conversely, sample participants showed lower adherence during weeks 5 and 6 (CALF exercise level 3 intensity) with an overall 53% (10/19) of the participants always adherent. Almost 50% more individuals in the FOOTFIT group (6/10, 60%) were not always adherent compared with the FOOTFIT+ group (3/9, 33%).

**Table 3:** Frequency distribution for adherence across all exercises by the level of intensity for FOOTFIT and FOOTFIT+ groups.

Conditioning activities for lower leg function exercise intensity level^a^		FOOTFIT (n=12)	FOOTFIT+ (n=12)	Difference in proportions^b^	95% CI
**Level 1, n (%)**				0	−0.4 to 0.4
	Always adherent	4 (33)	4 (33)		
	Not always adherent	8 (67)	8 (67)		
**Level 2, n (%)**				0.3	−0.1 to 0.7
	Always adherent	8 (73)	5 (45)		
	Not always adherent	3 (27)	6 (55)		
**Level 3, n (%)**				0.3	−0.2 to 0.7
	Always adherent	4 (40)	6 (67)		
	Not always adherent	6 (60)	3 (33)		

^a^Level 1: weeks 1+2, level 2: weeks 3+4, and level 3: weeks 5+6.

^b^Difference and 95% CIs obtained using the Newcombe risk difference method.

#### Exercise Duration

Overall there was an increase of approximately 6 seconds in median exercise duration between level 1 and level 3 exercises for the FOOTFIT but not the FOOTFIT+ group (Table 4).

**Table 4:** Mean (SD) and median (range) exercise duration in seconds by exercise level for FOOTFIT and FOOTFIT+ groups.

Conditioning activities for lower leg function exercise levels^a^		FOOTFIT	FOOTFIT+	Difference in medians (SE)^b^	95% CI
**Level 1 (weeks 1+2), (n=12;12)**				1.9 (6.5)	−11.7 to 15.4
	Mean (SD)	29.0 (9.8)	31.7 (18.9)		
	Median (range)	24.3 (18.5-46.0)	25.9 (16.1-82.9)		
**Level 2 (weeks 3+4), (n=11;11)**				0.3 (6.6)	13.5 to 14.0
	Mean (SD)	30.3 (17.6)	30.9 (20.7		
	Median (range)	21.7 (16.4-71.0)	21.4 (17.3-84.7)		
**Level 3 (Weeks 5+6), (n=10;9)**				4.0 (13.2)	23.3 to 31.2
	Mean (SD)	34.5 (18.4)	36.2 (25.7)		
	Median (range)	30.6 (17.8-74.6)	26.3 (19.8-99.1)		

^a^Level 1: weeks 1+2, level 2: weeks 3+4, and level 3: weeks 5+6.

^b^Difference in medians (SE) and 95% CIs obtained using quantile regression.

## Discussion

### Principal Findings

In our study, we investigated the feasibility of FOOTFIT that combined a foot-worn accelerometer BEAT with a mHealth app and evidence-based foot exercises CALF; in the FOOTFIT+ group, an additional patient-provider communication option (FOOTFIT+) was included. The intervention was developed to promote adherence to a 6-week progressive exercise program suited to the needs and preferences of minimally ambulatory older adults with VLUs. The results showed participants had problems using the accelerometer and app but were mostly adherent to the exercise protocol. Minimal gains were made in exercise intensity and duration in both groups. It is important to point out that the study focused on feasibility and was not powered to detect differences between groups.

Our one-on-one coaching and MI physical activity interventions found to be feasible in our previous studies guided the development of our mHealth foot-worn sensor and exercise app intervention. Exercise is recommended for patients with VLUs to enhance calf muscle pump function, which, in turn, improves lower extremity function and may aid in wound healing. Supervised and unsupervised programs that incorporate resistance, flexibility, and moderate-intensity aerobics can be safely performed by individuals with VLUs [8]. Other benefits of exercise include improved microvascular circulation of the lower limb [26,27] and improved wound healing [9,10]; however, a recent review of six randomized controlled trials to examine the effects of exercise on healing showed high-quality evidence is lacking to support healing [6]. Many of the studies reviewed targeted fairly high-functioning adults residing in the community; had multiple intervention types such as 10,000 steps, behavior modification, or community-based groups; included small sample sizes; and measured numerous variables with disparate measures, making comparisons to make and draw conclusions across studies. Our feasibility studies of the use of mHealth devices specifically targeted functionally impaired, minimally ambulatory individuals; tracked and provided feedback on progress; and delivered motivational messages and reminders to enhance adherence to exercise.

### Adherence

Many of the participants in our study had little or no previous experience with accelerometers or apps; thus, we anticipated lower adherence rates, given the difficulty with using the device and phone app. However, 100% of the participants were adherent to our 6-week exercise program more than 85% of the time in terms of duration, whereas 33% of the participants adhered more than 85% in terms of frequency of exercises. This finding is lower than findings of a 12-week home-based program in which 59% of individuals adhered to exercise more than 75% of the time [8]. Current evidence of mHealth interventions to promote physical activity demonstrate positive short-term effects (increased daily step counts and minutes spent on physical activity); however, evidence for long-term effects is lacking [28]. We recognize our results may have differed because of the short intervention period and may not have captured decreased sustainability over time. We also point out that this study differs from our previous studies in delivery modalities (face-to-face interaction with wound provider during a clinic visit or one-on-one visit with a coach via social media visit). It would be interesting to determine which modality, including the use of an app, is preferable/acceptable to patients and the barriers and facilitators to adoption of each approach.

Furthermore, at the time of accelerometer and app development, the system may not have been ideally designed for an older population. Although fitness and other exercise apps are developing at a rapid pace for waist and wrist-worn devices, to our knowledge, our accelerometer was the first triaxial method used to measure minute foot movements. Having to push a very small button on the accelerometer proved to be a challenge for the participants, many of whom had visual problems, difficulty with bending over, and toggling through the phone app. Many forgot to turn off the device, causing miscommunication with the app and draining the battery. A major problem with using the phone was the participants’ inability to quickly double click the home button to turn the app on and off.

App features such as reminders have been found to be essential for app engagement and are influenced by social demographics; health characteristics; intentions to change; and actual health behaviors, motivation, and support [29]. In a study of app use for physical activity, the findings suggested that older individuals, males, having a degree, or less than high school education are associated with reduced likelihood of adopting health apps [30]. The findings from a study of health apps in older adults demonstrated increased levels of physical activity compared with those without a device or health app. It remains unclear whether mHealth interventions have a greater impact on adherence to physical activity recommendations than non-mHealth interventions such as printouts.

### Provider Engagement

We anticipated greater user engagement of the patient-provider communication feature. Participants in the FOOTFIT+ group were aware that they could communicate via text, emails, or phone with the provider, and perhaps, this knowledge improved their adherence rates from 33% always adherent to CALF level 1 to 67% to CALF level 3 compared with FOOTFIT 33% and 40%, respectively. The reasons expressed by participants for not calling the wound provider included “I didn’t want to bother her,” “I could call the study coordinator if I had a question or problem,” and “I didn’t have any problems I needed to tell my doctor and if I did, I talked to her during my wound clinic visit.” The wound providers reported they did not have time to review the reports (these were compiled weekly by the study coordinator and sent directly via email to the providers) and noted they did not see the need to call participants as they thought they were doing well. In addition, the wound providers saw the participants weekly in their clinics and asked at that time how they were doing with the study. Thus, the patient-provider communication feature was viewed as not being necessary. However, in a longer study and without weekly clinic visits, this might have been a more useful option for patients to check in with their providers.

### Limitations and Strengths

The limitations of this randomized controlled trial include a small sample size, as the primary aim was feasibility study. In addition, because the accelerometer and app were specifically designed for individuals with VLUs, the generalizability of results is limited. However, other minimally ambulatory populations with chronic conditions that have limited function may benefit from this type of physical activity approach. We recognize the intervention did not include a specific adherence-enhancing component other than daily alerts; there were no provisions for behavioral change support. Behavioral approaches should be incorporated into future designs to enhance motivation and user engagement. In addition, our evidence-based CALF program was specifically designed for a progressive, short initial exercise *boost* for this minimally ambulatory population, prior to them undertaking a more rigorous physical activity program. Thus, our findings do not add to the fields of physical activity or exercise sciences in a way that advances our understanding of the influence of exercise on wound healing.

The strengths of the study include the use of a foot-worn device specifically designed for the population and the use of evidence-based exercises tailored to enhance foot function. The use of the RE-AIM as the feasibility framework provided both quantitative (number of log-ins) and qualitative (open-ended questions about usability) information and allowed for detailed participant perspectives and experiences using the intervention components. This mHealth study is unique to the best of our knowledge; it is the first study to focus on a minimally physically functional group of older adults to promote an exercise intervention focused solely on the foot and ankle.

### Conclusions

In this study, physically low-functioning adults with VLUs perceived the foot-based accelerometer BEAT, CALF exercises, and phone-based app (FOOTFIT) as somewhat acceptable and the added communication feature (FOOTFIT+) as not particularly useful. Most participants said they would perform the exercises without the accelerometers and reminders. As readily available apps continue to evolve and promote physical activity in populations with chronic conditions, our stand-alone intervention did not seem sufficient to promote exercise in this population. Future mHealth interventions should consider adding tailored adherence-enhancing components, such as added support (although the participants had access to the wound clinician) to positively influence behavior change, improve the functionality of the wearable device, and revise the app to make it more intuitive and bigger/easier to read on the phone and tablet. From our experience with three small trials, we advocate that providers such as wound specialists or primary care providers discuss patient preferences for engaging in the frequency and types of physical activity to enhance lower leg physical functioning.

## Appendix

Multimedia Appendix 1 CONSORT EHEALTH checklist (v 1.6.1).

## Abbreviations

BEAT: Bluetooth-enabled acceleration tracking
CALF: conditioning activities for lower leg function
CONSORT: Consolidated Standards of Research Trials
mHealth: mobile health
MI: motivational interviewing
RE-AIM: reach, effectiveness, adoption, implementation, and maintenance
VLU: venous leg ulcers
